# Differential Rearing Alters Forced Swim Test Behavior, Fluoxetine Efficacy, and Post-Test Weight Gain in Male Rats

**DOI:** 10.1371/journal.pone.0131709

**Published:** 2015-07-08

**Authors:** David L. Arndt, Christy J. Peterson, Mary E. Cain

**Affiliations:** Department of Psychological Sciences, Kansas State University, Manhattan, KS, United States of America; Radboud University, NETHERLANDS

## Abstract

Environmental factors play a key role in the etiology of depression. The rodent forced swim test (FST) is commonly used as a preclinical model of depression, with increases in escape-directed behavior reflecting antidepressant effects, and increases in immobility reflecting behavioral despair. Environmental enrichment leads to serotonergic alterations in rats, but it is unknown whether these alterations may influence the efficacy of common antidepressants. Male Sprague-Dawley rats were reared in enriched (EC), standard (SC), or isolated (IC) conditions. Following the rearing period, fluoxetine (10 or 20 mg/kg, i.p.) was administered 23.5 hrs, 5 hrs, and 1 hr before locomotor and FST measures. Following locomotor testing and FST exposure, rats were weighed to assess fluoxetine-, FST-, and environmental condition-induced moderations in weight gain. Results revealed an antidepressant effect of environmental enrichment and a depressant effect of isolation. Regardless of significant fluoxetine effects on locomotor activity, fluoxetine generally decreased swimming and increased immobility in all three environmental conditions, with IC-fluoxetine (10 mg/kg) rats and EC-fluoxetine (20 mg/kg) rats swimming less than vehicle counterparts. Subchronic 20 mg/kg fluoxetine also induced significant weight loss, and differential rearing appeared to moderate weight gain following FST stress. These results suggest that differential rearing has the ability to alter FST behaviors, fluoxetine efficacy, and post-stressor well-being. Moreover, 20 mg/kg fluoxetine, administered subchronically, may lead to atypical effects of those commonly observed in the FST, highlighting the importance and impact of both environmental condition and dosing regimen in common animal models of depression.

## Introduction

Depression detrimentally affects people’s lives, both mentally and physically. With mood disorders impacting millions worldwide, depression has undoubtedly become a major global health burden [1;2]. Both environmental factors and serotonergic neurotransmission are known to play key roles in the etiology of this debilitating disorder.

In the preclinical setting, the forced swim test (FST) is often used as a method of measuring the efficacy of antidepressants in rodents. During the FST, an increase of escape-directed behavior is believed to reflect an antidepressant-like state, whereas the presence of immobility presumably reflects a state of behavioral despair and depression [3]. Interestingly, while antidepressants increase escape-directed behaviors in the FST, they decrease general locomotor activity, and the inclusion of the locomotor test offers a positive control for the observed behavioral effects of antidepressants in the FST.

One widely used antidepressant, fluoxetine, is a selective serotonin reuptake inhibitor (SSRI) that was first developed and reported in 1974 [4] and has become one of the most commonly prescribed SSRIs. Since it was first reported, it has been extensively studied in rodent models of depression. When administered to rats at therapeutic doses and through modest regimens, fluoxetine should result in an increase in swimming and a decrease in immobility [3]. Research has also shown that the antidepressant effects of fluoxetine, as observed in the FST, are not only dose-dependent, but also differ when administered acutely or chronically [5].

Generally speaking, several low doses of fluoxetine are ineffective when administered acutely, but effective when administered chronically, usually a 14-day period [5]. For example, chronic administration of 1, 2 and 5 mg/kg fluoxetine can significantly reduce immobility, but when the same doses are administered acutely or sub-chronically (three injections over a 24-hr period) no antidepressant effect is observed [6].

From a pharmacological standpoint, fluoxetine is widely believed to exhibit its antidepressant effects by delaying the reuptake of the neurotransmitter serotonin (5-HT), resulting in more 5-HT residing in the synapse for a longer period of time [7;8]. Fluoxetine also alters the function of 5-HT_1A_ receptors. The 5-HT_1A_ is a receptor subtype that regulates the firing rate of serotonergic neurons [9] and is likely to change following chronic exposure to fluoxetine [10]. The 5-HT_1A_ receptor serves as an inhibitory autoreceptor of serotonin. Furthermore, due to its location in the somatodendritic region of the serotonergic neuron, 5-HT_1A_ receptors also mediate serotonin effects on neuronal firing, further functioning as heteroreceptors [11]. When 5-HT_1A_ receptors are chronically activated via repeated fluoxetine exposures, these inhibitory receptors are desensitized, leading to increased serotonin release in terminal regions [12].

Research has shown that differentially rearing rats in enriched (EC), isolated (IC), or standard (SC) environments during adolescence leads to both neurochemical and behavioral changes in the brain [13;14]. The differential rearing period of 30 days has become a standard rearing duration for rodents that yields profound, reproducible, and robust behavioral and neurochemical differences between EC and IC rats [14;15]. Rearing rats in an enriched environment during the post-weaning period can result in enhanced synaptic plasticity, as evidenced by the enlargement of synaptic boutons, higher density of dendritic spines, and other enhancements in synaptic transmission [14;16–19]. Not surprisingly, serotonergic neurotransmission is also altered during the rearing process as well [20]. Following the environmental enrichment period, rats have enhanced expression of the gene for the 5-HT_1A_ receptor in the hippocampus [21], an area of the brain heavily implicated in depression [22], suggesting more functional serotonergic regulatory mechanisms in EC rats. This may make EC rats more sensitive to serotonergic compounds such as SSRIs, and may make them more apt to exhibit these drugs’ antidepressant-like effects in the FST.

Of the few studies investigating environmental enrichment and FST outcomes, enrichment appears to produce antidepressant-like effects and increased serotonin concentrations in the prefrontal cortex compared to control and isolation-reared rats, and these findings correlate with behavioral performance in the FST [23]. The literature further suggests a possible rearing-induced EC and IC divergence in response to various SSRIs. For example, Raz and Berger [24] concluded that antidepressants, particularly SSRIs, may normalize or stabilize serotonin function and restore the potential behavioral deficits produced by isolation rearing. Furthermore, administration of the SSRI sertraline can reduce depressive-like states in enriched and standard-housed rats, but it appears to have little or no effect in rats reared in isolation [25]. These differences could reflect significant changes in serotonergic function between EC and IC rats brought about by the rearing process which may also influence the efficacy of fluoxetine. Although there is an abundance of literature investigating the effects of fluoxetine and other SSRIs on FST behavior in standard-housed rats, relatively little is known about how these well-established drugs exert their antidepressant-like effects in differentially reared rats, and the efficacy of fluoxetine in environmentally enriched rats is unknown.

Therefore, the present study determined if environmental enrichment alters not only initial FST behaviors, but also fluoxetine-induced performance in the FST. The effects of both fluoxetine (10 or 20 mg/kg) and differential rearing on locomotor and FST behaviors were examined to determine if EC, IC, or SC rats differed in how they responded to subchronic administration of fluoxetine. Furthermore, body weights were recorded daily directly before and after fluoxetine exposure (20 mg/kg) to assess any possible long-term effects of either fluoxetine or differential rearing on post-stressor well-being.

## Method and Materials

### Subjects and Environmental Conditions

Male Sprague-Dawley rats were ordered from Charles River Laboratories and arrived in the laboratory at 21 days of age. Rats were then randomly assigned to environmental conditions where they reared in the enriched (EC), isolated (IC) or standard (SC) condition for 30 days. EC rats were housed in a group of 10 in a large metal cage (60 x 120 x 45 cm) that was lined with bedding. Fourteen non-toxic objects (children’s toys, PVC pipe, etc.) were placed in the cage. Seven of the objects were changed daily and all of the toys were changed weekly. EC rats were also handled daily during scheduled toy changes. IC rats were housed individually in hanging metal cages (17 x 24 x 20 cm) that had wire mesh fronts and bottoms, and solid sides. IC rats did not have novel objects and were not handled during the 30 day rearing period. SC rats were housed in pairs in standard plastic shoebox cages (20 x 43 x 20 cm) to provide a known laboratory standard for comparison [26]. SC cages had bedding, wire tops, and these rats were handled weekly during scheduled cage changes. All rats were housed under a 12:12 hour light: dark schedule, and had ad libitum access to food and water throughout the experiment. Lights were on between 0700 and 1900 hours, with temperature maintained at 22°C and humidity ranging from 30–45%. This study was carried out in strict accordance with the recommendations in the Guide for the Care and Use of Laboratory Animals of the National Institutes of Health [26]. The protocol was approved by the University Research Compliance Office of Kansas State University and the Institutional Animal Care and Use Committee of Kansas State University (Protocol Number: 3244). All efforts were made to minimize suffering.

### Apparatus

Locomotor activity was measured by recording photobeam interruptions in a test chamber measuring 40.64 x 40.64 x 40.64 cm (Coulbourn Instruments, TruScan 2.01). The chambers had Plexiglas walls and a plastic tray floor covered with bedding.

For the FST, rats were placed individually into a glass cylinder measuring 20.32 cm diameter X 40.64 cm height containing water (25°C ± 1.0°C). Cylinder water was deep enough (30 cm) to ensure that the rats’ hind-paws could not touch the cylinder’s bottom. The swimming sessions consisted of a 15-min pretest, followed 24 hrs later by a 5-min test session. The dimensions, temperature, and further procedures are based on the original FST [27], the modified FST [28;29], and those described in a FST protocol review [3]. The dimensions of the modified FST were implemented in the current study to better detect the efficacy of selective serotonin reuptake inhibitors [5;29;30].

The FST sessions were recorded by a video camera from the side of the cylinder and the animals' escape directed behaviors (swimming and climbing) and immobility were later manually scored. An experimenter was present in the room at all times during the swim sessions. After successful completion of the FST, each rat was dried with a towel, placed in a warm enclosure, and then placed in a dry transport cage (20 x 43 x 20 cm) equipped with bedding, food, and water. EC and SC rats were placed back in their home cages only after their cage mates completed the FST to prevent any possible social transfer of fear [31] to FST-naïve rats that could influence their upcoming FST behaviors.

### Drugs and Dose Regimen

Fluoxetine hydrochloride (10 or 20 mg/kg, i.p.) was purchased from Sigma or obtained from the NIMH CSDSP and was administered 23.5 hrs, 5 hrs, and 1 hr before locomotor or FST measures. Based on previous literature, this subchronic drug regimen results in prolonged brain penetration of the drug under study, and mimics a state of subchronic drug exposure and continuously elevated drug concentrations in the rat [5]. The fluoxetine injection schedule used for the locomotor test was identical to that used in the FST. Fluoxetine hydrochloride was dissolved in sterile distilled water, which served as vehicle, in a volume of 1 ml/kg. Drug dissolving was aided through use of a vortex mixer and mild sonication.

### Experiments

The three experiments were split up in two cohorts each. Table 1 illustrates the experimental setup of these three experiments. Following Experiment II of the experiment, we were concerned our counterbalanced drug design between locomotor and FST measures could have served as a confounding variable (due to the possibility of residual drug effects in FST-vehicle rats previously injected with fluoxetine for the locomotor test). Furthermore, during Experiment II, significant weight reductions were observed in locomotor-fluoxetine rats, and this weight loss was still significant at the time of the FST (in which these same rats were to receive vehicle injections). To control for the possibility of lingering locomotor drug effects in eventual FST-vehicle rats, Experiment III of the experiment was conducted with a new group of rats, in which a locomotor test was not conducted prior to the FST. Therefore, all rats were drug naïve going into the FST.

**Table 1:** Experimental Design.

	PND 21–51	PND 52–53	PND 56–61	PND 57–91
Experiment I	30-day rearing EC or IC rats; 2 cohorts of 24 rats (n = 48)	Habituation and locomotor test	FST	
Experiment II	30-day rearing EC, IC, or SC rats; 2 cohorts of 30 rats (n = 60)	Habituation and locomotor test	FST	
Experiment III	30-day rearing EC, IC, or SC rats; 2 cohorts of 30 rats (n = 60)	No locomotor test	FST	Post-fluoxetine and FST weights recorded

Experimental design and sample sizes for enriched (EC), isolated (IC), and standard (SC) rats. Drug treatment for Experiment I (fluoxetine, 10 mg/kg, i.p.) and Experiment II (fluoxetine, 20 mg/kg, i.p.) between the locomotor test and the FST was counterbalanced. Experiment III fluoxetine was administered at 20 mg/kg, i.p. Experiment I drug treatment conditions: EC-fluoxetine (n = 12); EC-vehicle (n = 12); IC-fluoxetine (n = 12); IC-vehicle (n = 12). Experiment II drug treatment conditions: EC-fluoxetine (n = 10); EC-vehicle (n = 10); IC-fluoxetine (n = 10); IC-vehicle (n = 10); SC-fluoxetine (n = 10); SC-vehicle (n = 10). Drug treatment conditions for Experiment III were identical to Experiment II. Vehicle-treated and drug-treated rats were housed in the same cages (half vehicle-treated and half drug-treated) for enriched and standard conditions for all three experiments.

### Behavioral Procedure

#### Experiments I and II

Rats underwent the 30-day rearing period described above. After rearing, rats were habituated to the locomotor chambers for 15 min and then received three injections of fluoxetine (10 or 20 mg/kg) or vehicle using the regimen described above. 24 hrs later, rats were then tested in the locomotor chambers. Experiment I consisted of EC and IC rats only, whereas Experiment II consisted of EC, IC, and SC rats. 3–7 days after locomotor testing, rats underwent the 15 min pretest for the FST. Following the pretest, rats received the fluoxetine or vehicle injections three times before the 5-min test session.

#### Experiment III

Experiment III rats did not experience the locomotor test prior to the FST. Identical FST procedures to Experiments I and II were used in Experiment III. Following fluoxetine administration and FST exposure, rats in Experiment III were weighed daily to assess fluoxetine-, FST-, and environmentally-induced moderations in weight gain.

## Scoring and Data Analyses

### Locomotor Test

An analysis of variance (ANOVA) was performed to evaluate Drug treatment (fluoxetine or vehicle) and Environmental condition (EC, SC, or IC) on total distance traveled (cm) during the locomotor test. Significant interactions were probed using multiple comparison tests to compare the effect of drug within each environmental condition, and to compare environmental conditions within each drug treatment. All alpha levels were set at *p* < 0.05.

### Forced Swim Test

Behavior during the first five minutes of the pretest was scored to determine the effects of differential rearing on initial FST performance [32]. The entire 5-min FST session was scored to determine the effects of both differential rearing condition and drug treatment on FST performance. The first 5 min of the pretest session and the 5-min FST session were broken into 60 5-sec bins in which the predominant behavior during each 5-sec period was scored as swimming, climbing, or immobility. This totaled 60 scores for each rat in each session. Behavioral data were scored by researchers blind to experimental conditions. The dependent measure for all analyses was the expression of escape-directed behavior (swimming and climbing) and the behavioral despair measure of immobility.

To ensure there was an increase in immobility from the pretest to the test session, the pretest was compared to the test session through multiple factorial analyses of variance [3;32]. During the pretest and the FST sessions, to determine if significant differences existed in escape-directed behavior or immobility, one-way ANOVAs were used to separately test the between-subjects factors: rearing condition (EC, IC, or SC) and pharmacological group (fluoxetine or vehicle control). Dependent measures (swimming, climbing, and immobility) were scored and separate ANOVAs were conducted for each dependent variable. Significant main effects for pretest analyses were probed with multiple comparison tests using Tukey-Kramer adjustments to control for family-wise error rate (α_FER_ = 0.05).

Significant effects for the FST session analyses were probed using a Bonferroni adjustment to control for family-wise error rate (α_FER_ = 0.05). A Bonferroni adjustment, rather than Tukey-Kramer, was appropriate for these FST analyses given that not all of the possible pairwise comparisons were of interest in the current behavioral measure. Thus, for each of the following analyses with any significant effects, only a subset of all possible pairwise comparisons was investigated.

### Changes in Body Weight

To assess the long-term effects of rearing condition, fluoxetine, and FST exposure on post-stressor weight gain in Experiment III, a repeated-measures ANOVA was conducted with rearing condition (EC, IC, and SC) and pharmacological group (fluoxetine or vehicle control) as the between-subjects factors. Percent change in weight from baseline (weight preceding fluoxetine and FST exposure) over a period of 30 days served as the dependent measure.

## Results

### Experiment I: 10 mg/kg Fluoxetine with Locomotor Test

**Fig 1:**
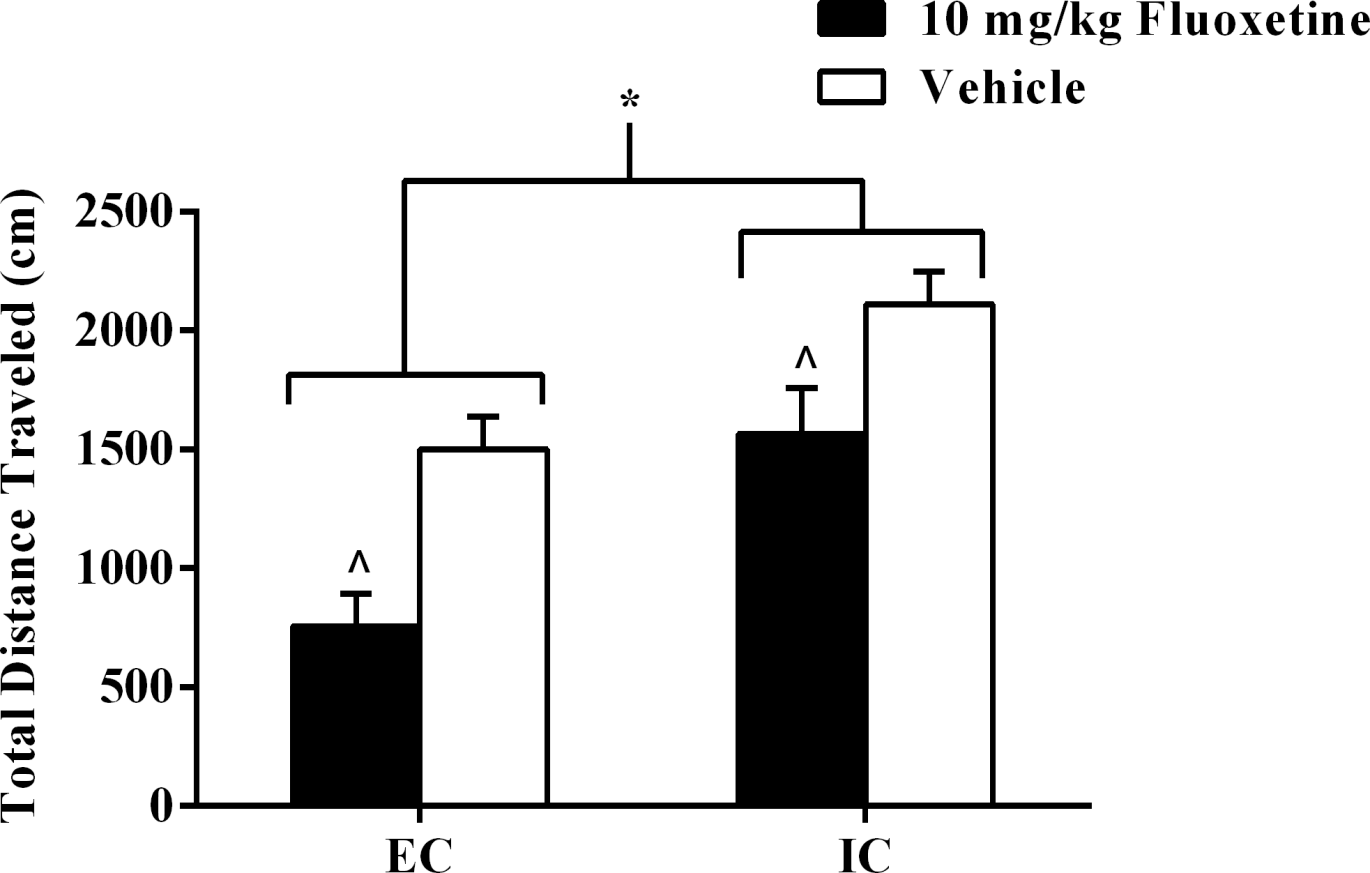
Experiment I Locomotor Activity following 10 mg/kg Fluoxetine. Experiment I total locomotor distance (cm) traveled (±SEM) in EC and IC rats after subchronic fluoxetine (10 mg/kg, i.p.) or vehicle administrations 23.5 hrs, 5 hrs, and 1 hr before the locomotor test. Asterisk (*) indicates that EC rats exhibited less locomotor activity than did IC rats. Caret signs (^) indicate that fluoxetine rats exhibited less locomotor activity than vehicle rats (*p* < .05).

The locomotor test revealed a significant main effect of both drug treatment, *F*(1, 20) = 17.70, *p* < .001, and environmental condition, *F*(1, 20) = 21.41, *p* < .001; EC rats exhibited less locomotor activity than did IC rats, and fluoxetine rats exhibited less locomotor activity than vehicle rats (*p <* .05; α_FER_ = .05; Fig 1).

#### Pretest analyses

There were no significant differences observed between EC and IC rats on pretest climbing, pretest swimming, or pretest immobility.

#### Test session analyses

For test session climbing and immobility behavior, there was not a significant main effect of drug treatment nor was there a significant main effect of environmental condition. There was also no significant interaction between the two in either dependent measure.

**Fig 2:**
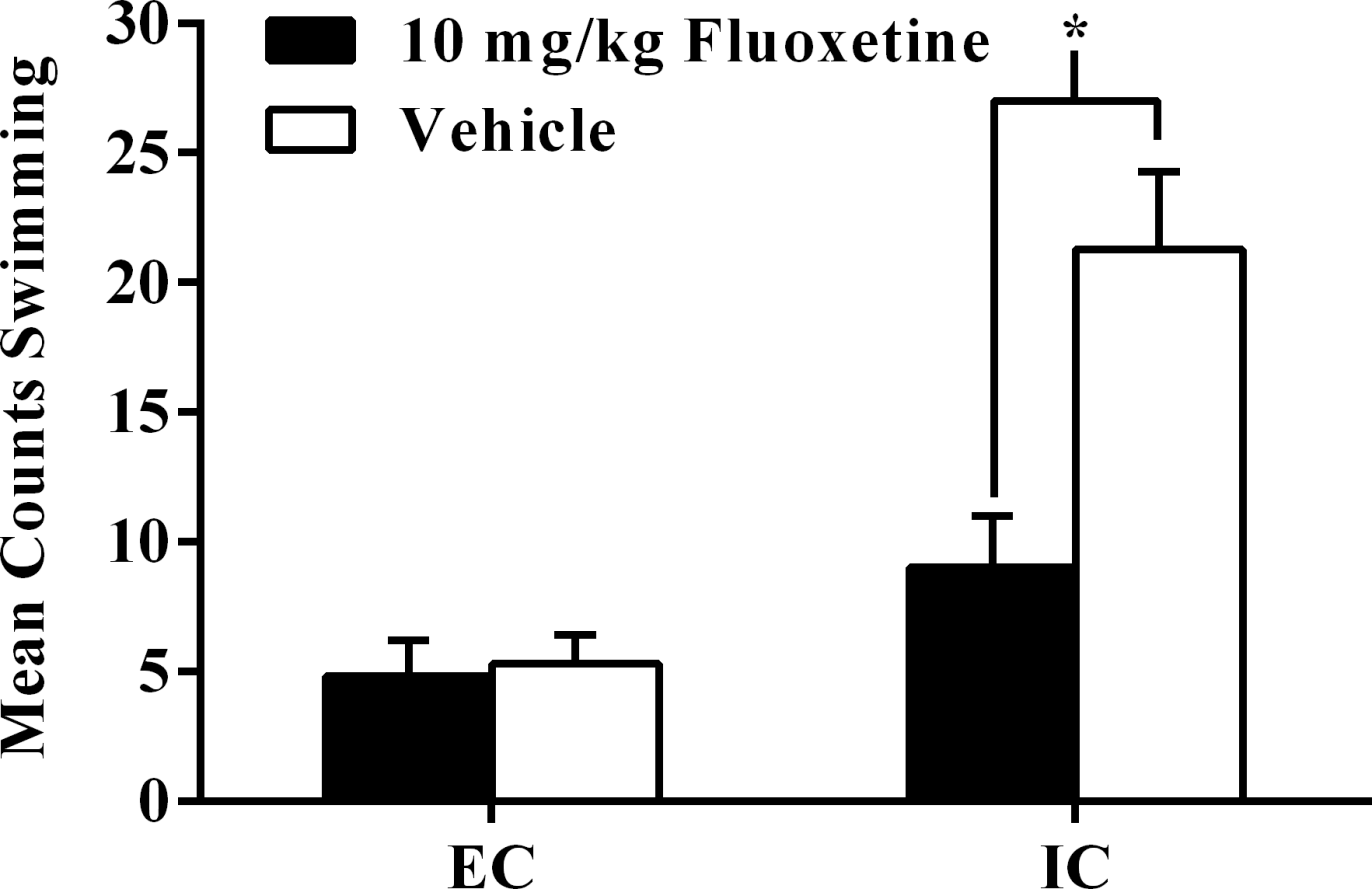
Experiment I Forced Swim Test Swimming. Experiment I total mean counts (±SEM) of swimming behavior between EC and IC rats after subchronic fluoxetine (10 mg/kg, i.p.) or vehicle administrations 23.5 hrs, 5 hrs, and 1 hr before the FST. Asterisk (*) indicates IC rats receiving vehicle exhibited significantly more swimming than IC rats receiving fluoxetine injections (*p* < .05).

Conversely, for test session swimming, there was a significant main effect of drug treatment, *F*(1, 44) = 9.82, *p* < .01. Vehicle rats exhibited more swimming in the FST test session than fluoxetine rats (*p <* .05; α_FER_ = .05). There was also a significant main effect of environmental condition, *F*(1, 44) = 24.78, *p* < .001, such that IC rats exhibited more swimming in the FST than EC rats (*p <* .05; α_FER_ = .05). Both of these main effects were qualified by a significant drug treatment x environmental condition interaction, *F*(1, 44) = 8.58, *p* < .01. IC-vehicle rats displayed more swimming in the test session than IC-fluoxetine rats (*p <* .05; α_FER_ = .05; Fig 2). However, there was no effect of drug in EC rats.

#### Behavioral changes from pretest to test session

To test if there were any differences in rats’ climbing, swimming, or immobility behaviors between the pretest and the FST session, and to determine whether environmental condition had any moderating effect on the session differences, three two-way ANOVAs were conducted. For each of the three FST behaviors (climbing, swimming, and immobility), a 2 (environmental condition: EC vs. IC) x 2 (session: pretest vs. test session) mixed-factorial ANOVA was conducted.

There was a main effect of session on climbing behavior, *F*(1, 46) = 52.46, *p* < .001, such that rats were climbing more in the pretest than they were in the test session. There was no significant main effect of environmental condition, and there was furthermore no significant interaction between environmental condition and session.

For swimming behavior, there was a main effect of session, *F*(1, 46) = 6.29, *p* < .05; rats were swimming significantly more in the pretest compared to the test session. There was also a significant main effect of environmental condition, *F*(1, 46) = 5.60, *p* < .05. EC rats exhibited more swimming than IC rats (*p <* .05; α_FER_ = .05). Both of these significant main effects were qualified by a significant session x environmental condition interaction, *F*(1, 46) = 17.65, *p* < .001. EC rats exhibited a significant decrease in swimming from the pretest to the test session (*p <* .05; α_FER_ = .05); however, IC rats did not significantly differ in their swimming behavior from the pretest to the test session.

There was also a significant main effect of session on immobility, *F*(1, 46) = 71.53, *p* < .001, such that rats displayed more immobility in the test session than in the pretest. There was also a significant interaction between session and environmental condition, *F*(1, 46) = 9.91, *p* < .05. Both IC and EC rats showed a significant increase in immobility from the pretest to the test session, and the magnitude of this increase was greater in EC rats compared to IC rats (*p <* .05; α_FER_ = .05).

Therefore, the aforementioned analyses confirmed that the pretest did indeed facilitate immobility and decrease escape-directed behaviors in the test session, as is commonly observed in FST studies. Additionally, when investigating the change in behavior from the pretest to the FST, EC rats were inclined to swim more than IC rats, suggesting an antidepressant effect of enrichment.

### Experiment II: 20 mg/kg Fluoxetine with Locomotor Test

**Fig 3:**
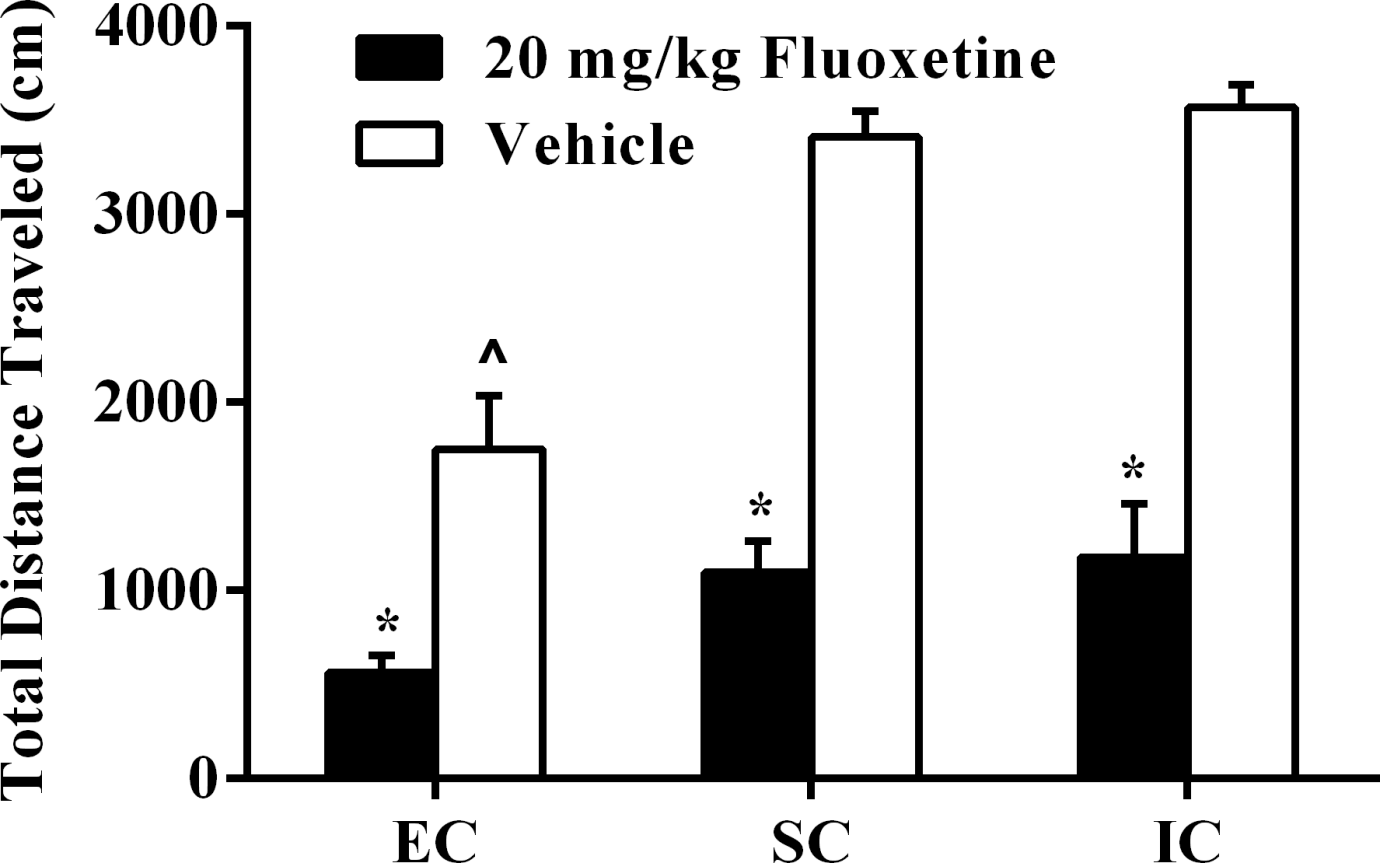
Experiment II Locomotor Activity Following 20 mg/kg Fluoxetine. Experiment II total locomotor distance (cm) traveled (±SEM) in EC, SC, and IC rats after subchronic fluoxetine (20 mg/kg, i.p.) or vehicle administrations 23.5 hrs, 5 hrs, and 1 hr before the locomotor test. Asterisks (*) indicate significant reductions in locomotor activity in fluoxetine-treated rats compared to vehicle controls for all three environmental conditions. Caret sign (^) indicates significant reductions in locomotor activity in EC-vehicle rats compared to both SC- and IC-vehicle rats (*p* < .05).

The locomotor test revealed a significant main effect of drug treatment, *F*(1, 54) = 151.59, *p* < .001, such that rats administered fluoxetine traveled significantly less than rats administered vehicle control injections. Results also revealed a significant main effect of environmental condition, *F*(2, 54) = 23.57, *p* < .001. As seen in Fig 3, fluoxetine decreased the total distance traveled in all three environmental conditions, *Fs*(1, 54) = 18.48–74.86, *ps* < .01.

#### Pretest analyses

**Fig 4:**
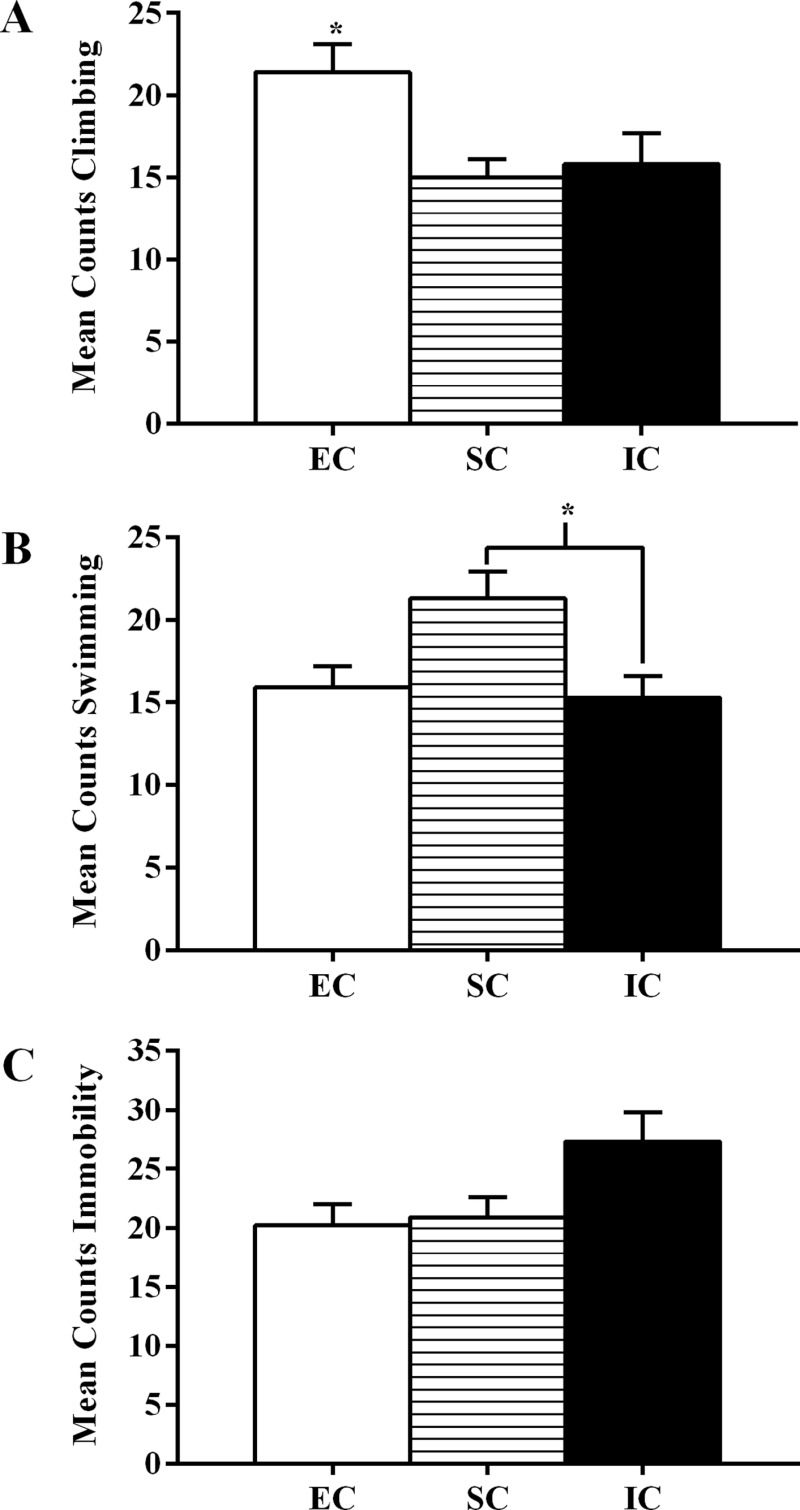
Experiment II Pretest Swimming, Climbing, and Immobility. Experiment II total mean counts (±SEM) of **(A)** climbing, **(B)** swimming, and **(C)** immobility behavior between EC, SC, and IC rats during the first five minutes of the forced swim pretest. **(A)** Asterisk (*) indicates EC rats exhibited significantly more climbing than both SC and IC rats during the first five minutes of the pretest. (**B)** Asterisk (*) indicates IC rats exhibited significantly less swimming than SC rats during the first five minutes of the pretest (*p* < .05).

For pretest climbing, there was a significant main effect of environmental condition, *F*(2, 55) = 4.66, *p* = .014. EC rats displayed significantly more climbing during the pretest than IC rats and SC rats (*p <* .05; α_FER_ = .05; Fig 4A), which again suggests an antidepressant effect of enrichment.

There was also a significant main effect of environmental condition on swimming during the pretest, *F*(2, 55) = 3.77, *p* = .029. IC rats exhibited significantly less swimming than SC rats (*p <* .05; α_FER_ = .05; Fig 4B), suggesting isolation rearing and its ability to augment depressive-like states compared to standard-housed rats.

Additionally, there was a significant main effect of environmental condition on rats’ pretest immobility, *F*(2, 55) = 3.57, *p* = .035 (Fig 4C). However, none of the environmental conditions were significantly different from each other. Regardless, when taken together, these pretest analyses suggest a possible antidepressant effect of environmental enrichment and a trend suggesting that isolation may elicit depressive-like states during initial FST measures.

#### Test session analyses

**Fig 5:**
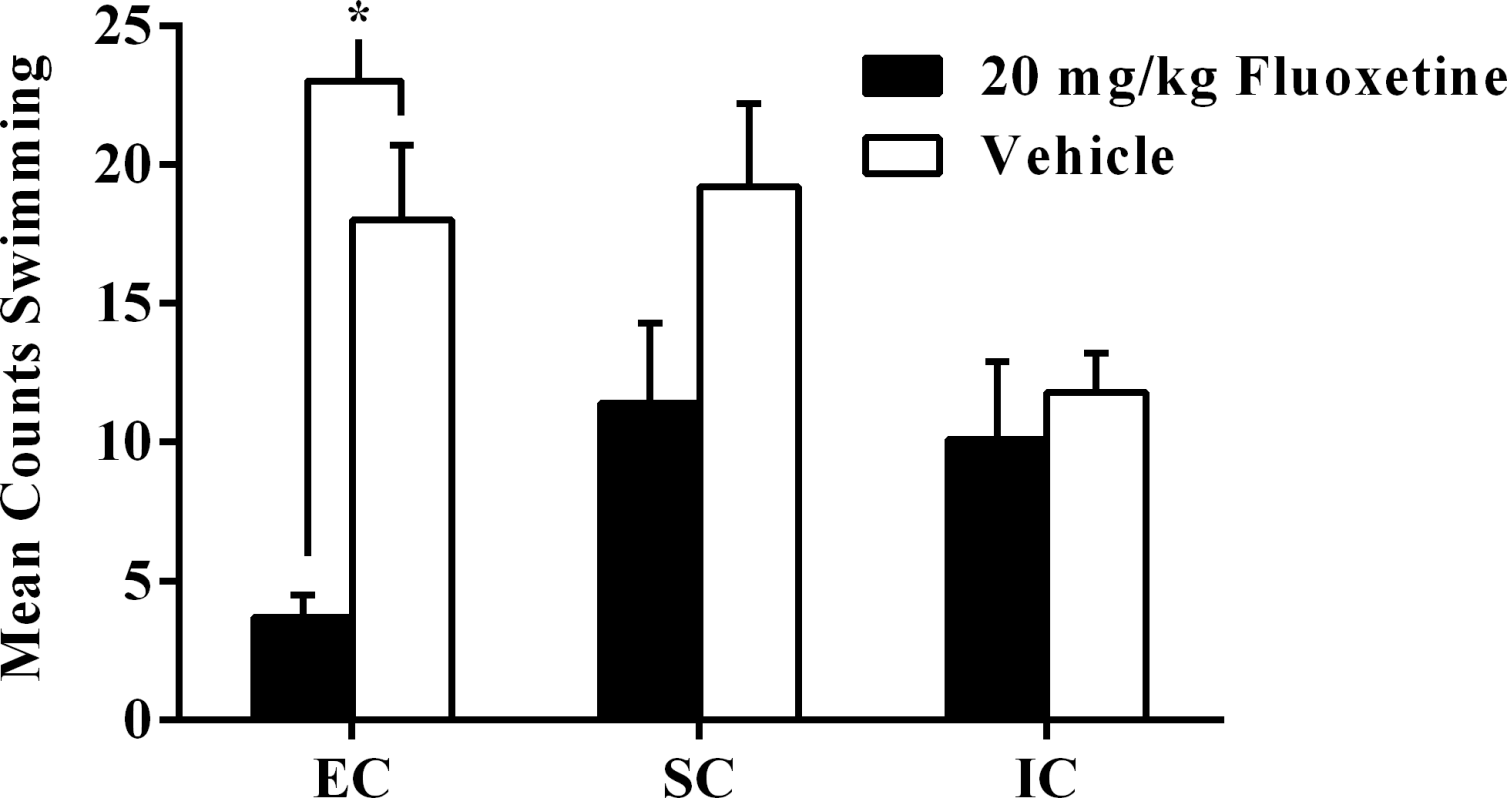
Experiment II Forced Swim Test Swimming Behavior Experiment II total mean counts (±SEM) of swimming behavior between EC, SC, and IC rats after subchronic fluoxetine (20 mg/kg, i.p.) or vehicle administrations before the FST. Asterisk (*) indicates EC rats receiving fluoxetine exhibited significantly less swimming than EC rats receiving vehicle injections (*p* < .05).

Results revealed a significant main effect of drug treatment on swimming, *F*(1, 51) = 16.41, *p* < .001. However, there was no significant main effect of environmental condition on rats’ swimming behavior. Rats treated with vehicle control injections displayed significantly more swimming than rats treated with fluoxetine. There was also a significant interaction between environmental condition and drug treatment on rats’ swimming in the test session, *F*(2, 51) = 3.46, *p* = .039. Multiple comparison tests indicated EC-fluoxetine rats swam significantly less than EC-vehicle rats (*p <* .05; α_FER_ = .05; Fig 5).

There were no significant differences in climbing or immobility in rats receiving fluoxetine or rats receiving vehicle in any of the three environmental conditions. It appears that 20 mg/kg fluoxetine administered subchronically induces atypical effects compared to those commonly observed in FST swimming measures.

#### Behavioral changes from pretest to test session

A repeated-measures ANOVA with repeated measures on session was run to compare changes in climbing behavior from the pretest to the test session. The analysis revealed a significant main effect of session, *F*(1, 55) = 18.14, *p* < .001. Rats exhibited significantly more climbing in the first five minutes of the pretest compared to their climbing behavior in the test session. An identical repeated-measures ANOVA on swimming behavior revealed a significant main effect of session, *F*(1, 55) = 20.39, *p* < .001, such that rats swam significantly more in the pretest than they swam in the test session. Similar to the effect on climbing, this suggests that rats were less likely to display the escape-directed behavior of swimming in the test session compared to the pretest. Another repeated-measures ANOVA also revealed a significant main effect of session on immobility, *F*(1, 55) = 109.71, *p* < .001, such that rats displayed significantly more immobility in the test session compared to their immobility in the pretest, illustrating the FST’s ability in the current study to facilitate immobility in the test session via the pretest.

### Experiment III: 20 mg/kg Fluoxetine without Locomotor Test

#### Pretest analyses

**Fig 6:**
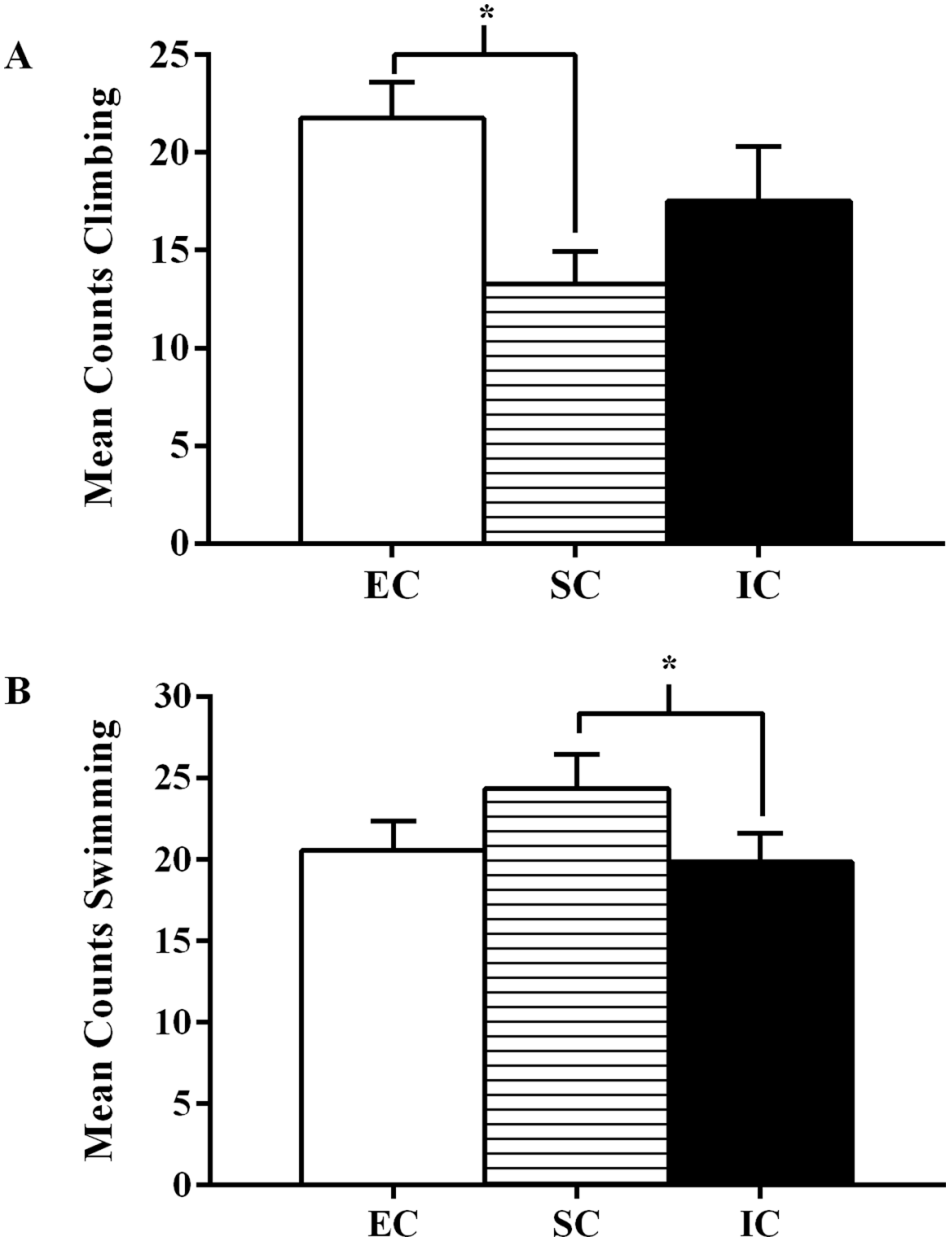
Experiment III Pretest Climbing and Swimming. Experiment III total mean counts (±SEM) of **(A)** climbing and **(B)** swimming behavior between EC, SC, and IC rats during the first five minutes of the forced swim pretest. **(A)** Asterisk (*) indicates EC rats exhibited significantly more climbing than SC rats during the first five minutes of the pretest. **(B)** Asterisk (*) indicates IC rats exhibited significantly less swimming than SC rats during the first five minutes of the pretest (*p* < .05).

There was a main effect of environmental condition on climbing in the pretest, *F*(2, 57) = 7.82, *p* = .001. EC rats climbed significantly more than SC rats, (*p <* .05; α_FER_ = .05; Fig 6A), once again displaying more escape-directed behaviors and suggesting an antidepressant effect of enrichment compared to SC rats.

There was also a significant difference between environmental conditions on rats’ pretest swimming scores, *F*(2, 57) = 3.38, *p* = .041. Similar to Experiment II results, IC rats displayed significantly less swimming than SC rats (*p <* .05; α_FER_ = .05; Fig 6B), suggesting again that isolated rats may be more prone to display fewer escape-directed behaviors.

Although there were no significant differences between environmental conditions in terms of pretest immobility, *F*(2, 57) = 2.61, *p* = .082, there was a trend toward EC rats displaying the least amount of immobility in the pretest compared to both IC and SC rats.

#### Test session analyses

**Fig 7:**
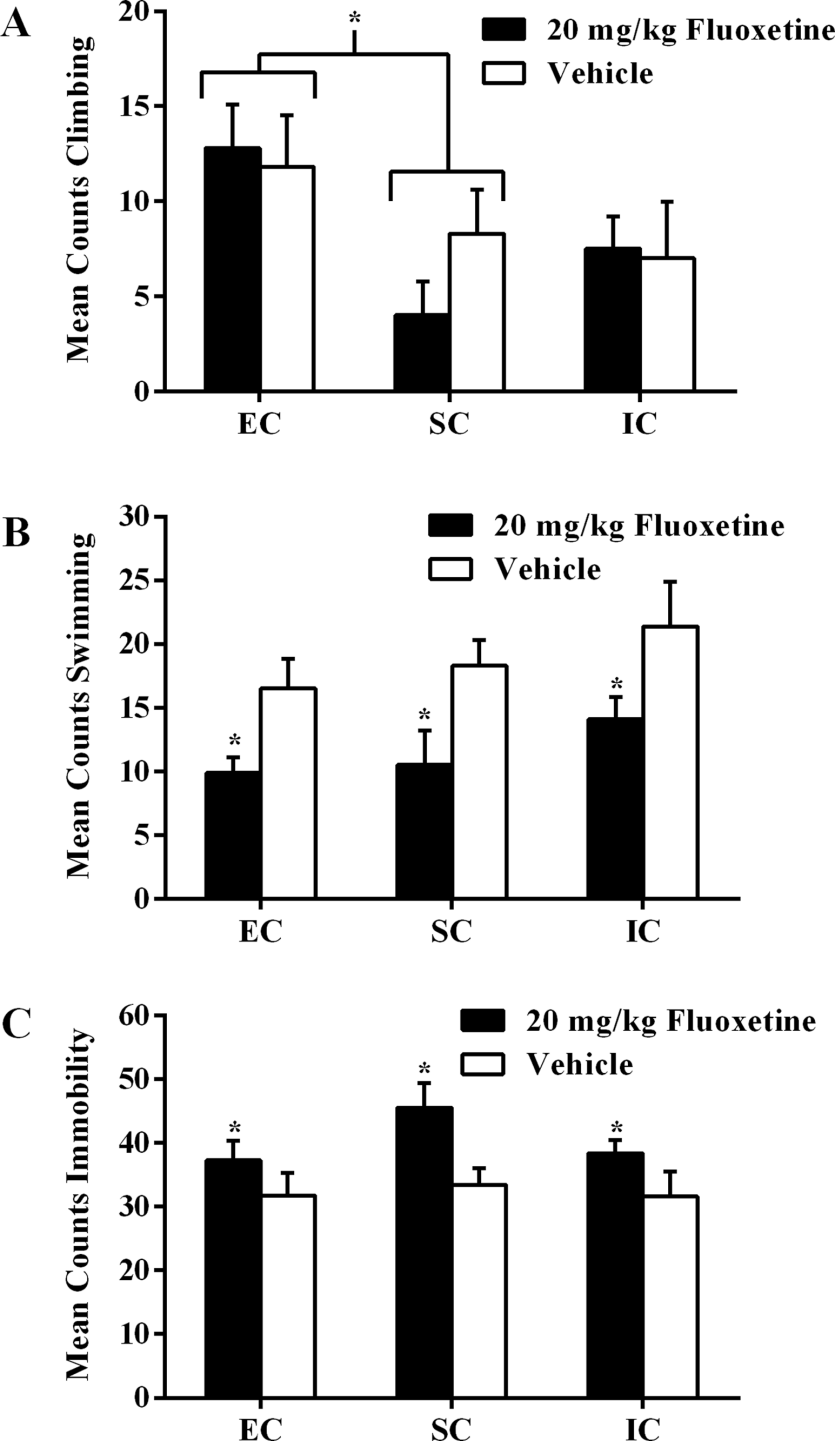
Experiment III Forced Swim Test Climbing, Swimming, and Immobility. Experiment III total mean counts (±SEM) of **(A)** climbing, **(B)** swimming, and **(C)** immobility behavior between EC, SC, and IC rats after subchronic fluoxetine (20 mg/kg, i.p.) or vehicle administrations before the FST. **(A)** Asterisk (*) indicates that EC rats exhibited more climbing behavior than SC rats, irrespective of drug treatment. **(B)** Asterisks (*) indicate that fluoxetine rats exhibited less swimming behavior than vehicle control rats. **(C)** Asterisks (*) indicate that fluoxetine rats exhibited significantly more immobility than rats administered vehicle control injections (*p* < .05).

There was no main effect of drug treatment on climbing behavior, but there was a main effect of environmental condition, *F*(2, 54) = 3.91, *p* < .05. EC rats climbed significantly more than SC rats (*p <* .05; α_FER_ = .05; Fig 7A). Furthermore, there was a significant main effect of drug treatment on swimming, *F*(1, 54) = 14.14, *p* < .001. Rats that received fluoxetine exhibited less swimming than vehicle rats (*p <* .05; α_FER_ = .05; Fig 7B). Lastly, drug treatment had a significant main effect on rats’ FST immobility, *F*(1, 54) = 9.17, *p* < .01; fluoxetine rats exhibited more immobility than vehicle rats (*p <* .05; α_FER_ = .05; Fig 7C), once again illustrating the atypical drug effect of fluoxetine at this current dose and regimen.

#### Behavioral changes from pretest to test session

Consistent with the previous experiments, the repeated measures ANOVA revealed that in the pretest, rats climbed and swam more, and displayed less immobility compared to their behavior in the test session (*F*s(1,57) = 27.69–120.07, *p*s < .05). For climbing and immobility, environmental condition did not moderate the effect of session (both *p*s > .05); however, environmental condition did moderate the effect of session for rats’ swimming behaviors, *F*(2, 57) = 3.53, *p* < .05, such that EC and SC rats swam more in the pretest compared to the test session (both *p*s < .01; α_FER_ = .05), but IC rats did not differ in their swimming behavior between sessions (*p* > .05). This was a similar finding compared to Experiment I rats in that IC rats did not alter their swimming behaviors from the pretest to the test session.

#### Changes in body weight gain

In order to examine if environmental condition and drug treatment influenced rats’ weight gain or loss following drug treatment or the FST, rats’ percentage weight change from baseline was recorded for 30 days post-fluoxetine and FST exposure. To analyze whether rats’ weights differed over time due to either environmental condition or drug treatment, a 3-way repeated-measures ANOVA was conducted. Weight differences were observed between the two cohorts in Experiment III. When rats were separated by environmental group and drug treatment, cohort I rats differed over time in their weights from cohort II rats, *F*(56, 1232) = 4.08, *p* < .001. In all three experiments, the two cohorts in each experiment were compared to each other to ensure they did not differ in swimming, climbing, or immobility for both the pretest and FST sessions. Indeed, no considerable differences existed between the two cohorts in any FST measure. Thus, it was deemed justifiable to combine the cohorts’ data for the FST in all experiments, while separating the cohorts for the weight data in Experiment III.

**Fig 8:**
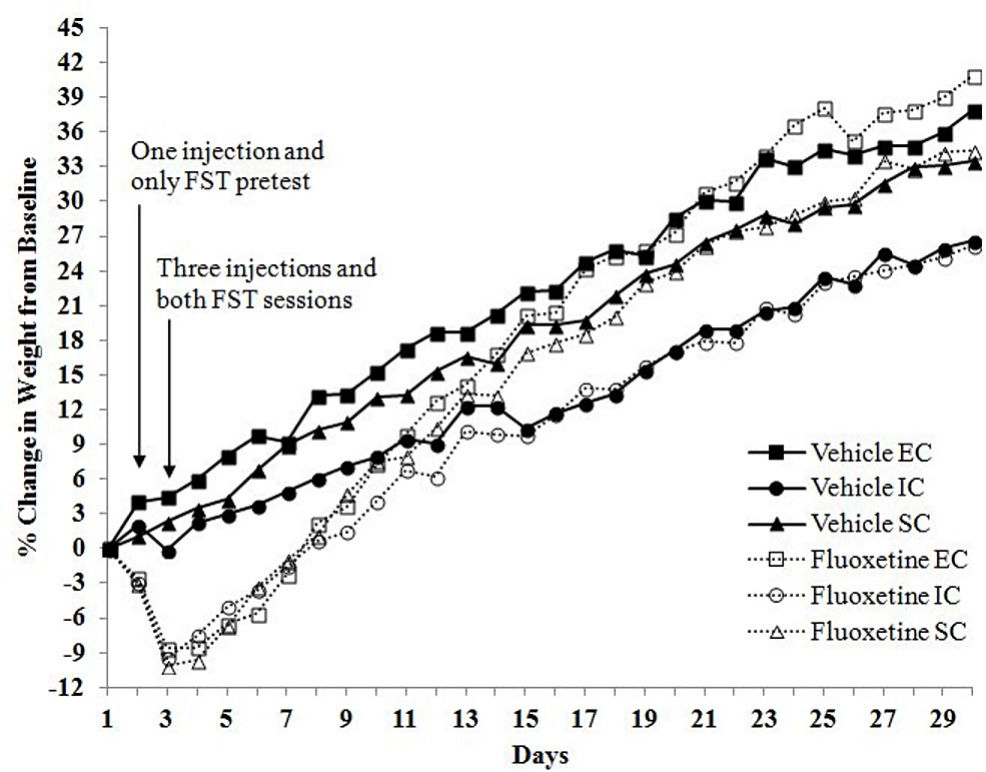
Percent Change in Body Weight. Percent change in body weight from baseline after FST and subchronic fluoxetine (20 mg/kg, i.p.) in EC, IC, and SC rats in cohort I. Day 1 reflects body weights before fluoxetine and FST exposure. The significant interaction between drug and environmental condition on rats' percent change in weight depends on the time that has passed since fluoxetine or vehicle administration (*p* < .05).

As illustrated in Fig 8, there were significant main effects of environmental condition, *F*(2, 21) = 38.34, *p* < .001, and drug treatment, *F*(1, 21) = 12.73, *p* < .05, on body weight gain in cohort I rats. Furthermore, all of these main effects are qualified by a three-way (days x environmental condition x drug treatment) interaction, *F*(62, 651) = 1.87, *p* < .001. Specifically, the interaction between drug and environmental condition on rats' percentage change in body weight depends on the time that has passed since fluoxetine or vehicle administration, with EC rats displaying more weight gain from baseline following the FST.

**Fig 9:**
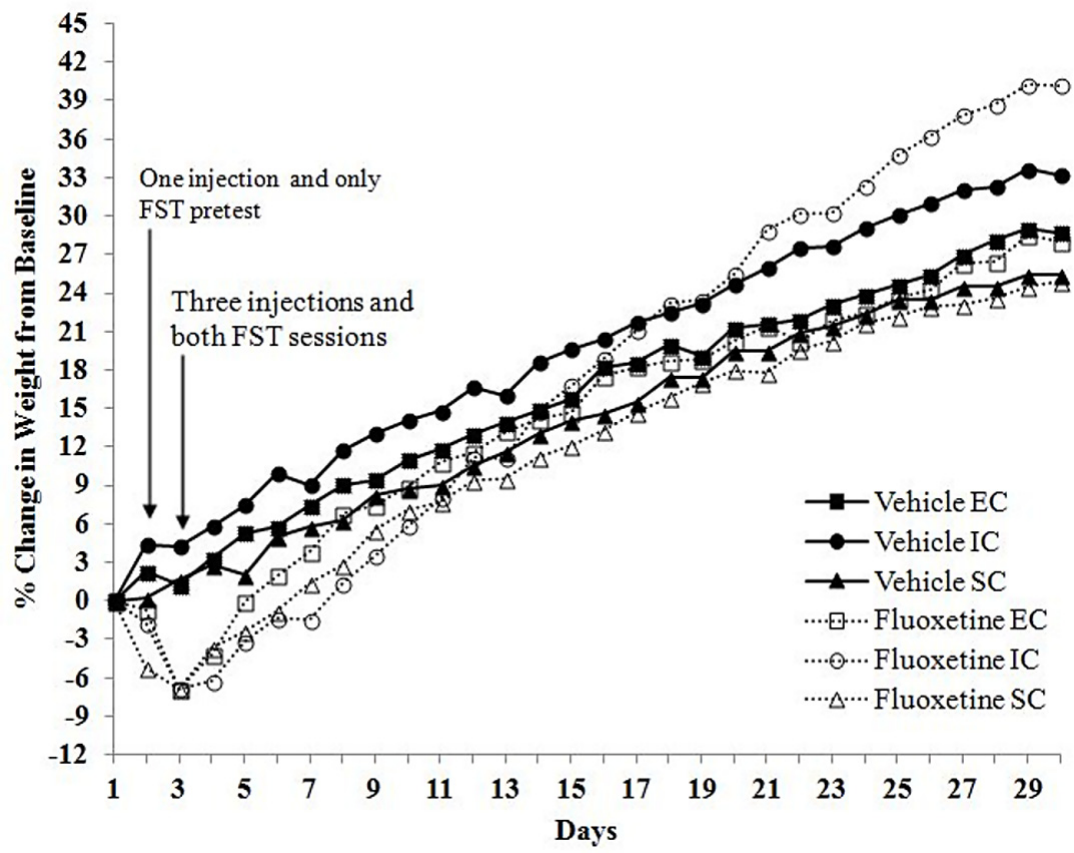
Percent Change in Body Weight. Percent change in body weight from baseline after FST and subchronic fluoxetine (20 mg/kg, i.p.) in EC, IC, and SC rats in cohort II. Day 1 reflects body weights before fluoxetine and FST exposure. The significant interaction between drug and environmental condition on rats' percent change in weight depends on the time that has passed since fluoxetine or vehicle administration (*p* < .05).

Compared to cohort I rats, analyses also revealed a three-way (days x environmental condition x drug treatment) interaction for cohort II rats, *F*(62, 713) = 2.91, *p* < .001; however, this interaction followed a different pattern. As can be seen in Fig 9, although the interaction between drug and environmental condition on rats' percentage change in weight depends on the time that has passed since fluoxetine or vehicle administration, it appears that IC rats are gaining more weight from baseline compared to EC rats.

Regardless of the effect of rearing condition, it should be noted that the subchronic fluoxetine administered in the current study, both at 10 and 20 mg/kg, i.p., resulted in significant weight loss in the majority of fluoxetine-treated rats. Fluoxetine-treated rats displayed visible fatigue, which may have been due to decreased food intake and depleted energy levels prior to FST measures. Nonetheless, results suggest that differential rearing has the ability to moderate post-stressor and/or post-fluoxetine weight gain.

## Discussion

The current study provides support that 30 days of differential rearing is enough to elicit differences in not only initial FST pretest behaviors, but also changes in fluoxetine efficacy and post-stressor moderation of weight gain. To our knowledge, this is the first experiment to show differences in rearing-induced moderations in weight gain following FST exposure. The current study also provides evidence that differential rearing and fluoxetine regimen, along with dose, can significantly impact FST results.

Many factors can influence performance in the forced swim test [33]. In the current study, rodent tails would occasionally touch the bottom of the cylinder, particularly during rare diving events or during the beginning of the forced swim session when rats were inclined to display vigorous escape-directed behavior. However, it was not evident that rats were using their tails for buoyancy support. Touching the bottom of the cylinder with either tails or hind paws could certainly influence the outcome of FST results. Therefore, the current experiments used a water depth of 30 cm as recommended [3] to prevent potential confounded results.

In the current study, for the initial behavioral test of locomotor activity, rats reared in an enriched environment displayed less locomotor activity than standard and isolated rats. These results are consistent with previous literature in that EC rats display less locomotor activity compared to rats reared in either isolated or standard conditions [34;35]. Rats were habituated to the locomotor apparatus one day before testing, which reduced the likelihood of novelty as an explanation for the behavioral differences observed between enriched, isolated, or standard-housed rats. Moreover, the locomotor test also revealed that fluoxetine significantly decreased locomotor activity in all three environmental conditions. This well-established effect [3] highlights the different neural substrates underlying locomotion compared to FST behavior. Importantly, the injection regimen of fluoxetine administered before the locomotor test (23.5 hrs, 5 hrs, and 1 hr beforehand) matched the regimen administered to rats before the FST. This significant decrease in locomotor activity after fluoxetine administration confirmed a robust drug effect in all three environmental conditions. Thus, the drug effect in the locomotor test highlights the paradoxical FST findings in that fluoxetine, at this regimen and dose, led to a decrease in swimming behaviors. This is contrary to what is typically observed with lower doses through chronic administrations.

Other than these differences observed during locomotor testing, in both Experiment II and Experiment III, EC rats, in the absence of fluoxetine, displayed significantly more climbing behavior, suggesting that EC rats were more inclined to display antidepressant-like states (climbing) when faced with an immediate stressor such as the FST. These findings are generally consistent with previous literature showing that enrichment can provide a protective effect against depressive-like states [32;36;37], whereas isolation rearing has been shown to augment the expression of depressive behaviors [38].

The antidepressant evidence of environmental enrichment in the current study also supports preexisting literature suggesting enrichment-induced neuronal alterations that mimic serotonin transporter (SERT) inhibition. For example, environmental enrichment with voluntary exercise can lead to the reduction of tryptophan-hydroxylase (TPH)-immunoreactive neurons, and these reductions are similar in magnitude to what is observed in fluoxetine-treated rats following seven days of fluoxetine treatment [39]. TPH is one of the enzymes responsible for the synthesis of serotonin [40], and is found to be increased in postmortem tissue of treatment-naïve suicide victims [41] which implies that TPH works as a possible stimulatory response to compensate for low 5-HT levels in depression [42]. The findings in the current study, albeit implementing a different fluoxetine treatment regimen, suggest that some of the antidepressant effects of both enrichment and SSRIs may be acting through common neural mechanisms.

Furthermore, the prefrontal cortex (PFC) appears to play an integral role in the neural circuitry of depression [43]. For instance, many established antidepressant drugs increase dorsal PFC activity and decrease ventral PFC activity [44]. Because enriched and isolated rats differ in PFC functionality [20], the benefits of enrichment observed in FST behavior could stem from enhanced serotonergic function in the PFC [23;45].

Despite the antidepressant effect of enrichment in the current study, the preexisting literature regarding environmental enrichment on FST performance remains inconsistent and unclear, from having no effect [46], to the production of antidepressant-like states [23;32]. Cui et al. [46] did not find an antidepressant-like effect of enrichment after 30 days of rearing during postnatal days 22–52. Brenes et al. [23] observed that enriched rats displayed decreased immobility and increased escape-directed behavior compared to IC or SC rats, but the rats were housed in their respective environmental conditions for 84 days prior to the FST. Brenes et al [32] then implemented a shorter rearing period and found that SC and IC rats displayed increased immobility from the pretest to the test session, but EC rats did not display a significant change in immobility between the two tests. Compared to isolates, it has been found that rats reared in an enriched environment for six weeks display increased escape-directed (antidepressant) behavior and decreased immobility (depressive behavior) in the FST [47]. Therefore, perhaps there are critical enrichment-induced changes during adolescence that enable enrichment to decrease depressive-like behaviors in the FST. The present study provides support of the ability of the 30-day enrichment period to reduce depressive behaviors during initial FST periods.

In addition to the antidepressant effects of enrichment, when comparing behavioral changes from the pretest to the test session in Experiment I and Experiment III rats, two similar session X environmental condition interactions were observed. In Experiment I, EC rats exhibited decreased swimming from the pretest to the test session, whereas IC rats exhibited slightly more swimming. Likewise, in Experiment III rats, we observed a similar interaction, in that EC and SC rats’ swimming significantly decreased from the pretest to the test session, whereas IC rats did not significantly decrease their swimming. This suggests that the ability of the pretest to facilitate the lack of escape-directed behavior in the test session appears to be blunted in IC rats, whereas EC and SC rats may realize, to a better extent than IC rats, that they are in an environment where escape is not possible, and thus choose not to expend energy by swimming. This reoccurring finding in both Experiment I and Experiment III rats adds to the behavioral complexities and differences between EC and IC rats and their responses to FST exposure and stressors.

In addition to rearing-induced changes to pretest behaviors, the current study indicates that differential rearing alters the efficacy of fluoxetine as well. Overall, the current dosing regimen appears to be reversing the effects typically observed in fluoxetine FST studies, such that fluoxetine generally reduced swimming and increased immobility. The observed swimming effect may not only be dose-dependent, but also condition-dependent. Specifically, fluoxetine led to a decrease in swimming in IC rats following 10 mg/kg, but a decrease in EC rats following the 20 mg/kg dose. Regardless of the environmental condition differences, fluoxetine at this regimen still led to significant reductions in antidepressant-like states. It remains unclear which specific neurochemical differences between EC and IC rats may be responsible for these varying decreases in swimming behaviors between the two doses.

Neurochemical analyses conducted by Brenes and colleagues [23] have shown that after 84 days of differential rearing, EC rats, compared to IC and SC rats, have enhanced 5-HT concentrations in the hippocampus and frontal cortex which correlates positively with swimming and negatively with immobility in the FST. This suggests that longer rearing periods may be accompanied by additional neurochemical changes that could impact subsequent behavioral measures such as the FST. One of these additional changes seen in EC rats may manifest in the form of enhanced neurogenesis compared to IC and SC rats. Interestingly, fluoxetine treatment and environmental enrichment have both been shown to augment cell-proliferation and cell survival within the hippocampus [48]. This may be one of the neurochemical mechanisms responsible for the alteration of fluoxetine’s efficacy between environmental conditions following the differential rearing period.

It is important to note that the doses of fluoxetine administered in the current study (10 and 20 mg/kg) have previously elicited antidepressant outcomes in the FST. Significant reductions in immobility have been found using a smaller dose (5–10 mg/kg), even when the smaller doses were administered subchronically [49–51]. At the 20 mg/kg dose, there have been instances in which fluoxetine led to reductions in immobility [52;53]. However, similar to the current study, Jang et al. [54] observed that 20 mg/kg fluoxetine given three times in a 24-hr period prior to the FST failed to increase swimming. Therefore, literature suggesting robust antidepressant effects (increased escape-directed behavior and decreased immobility) following subchronic, i.p. administration of fluoxetine is lacking.

Many studies have been conducted involving the effects of fluoxetine in adult rodents, and although fluoxetine is approved for use in children, preclinical findings suggest that the efficacy of fluoxetine differs in the developing rodent brain [10]. Evidence suggests that the antidepressant efficacy of fluoxetine may not only be age-dependent, but may also depend on levels of plasticity and neurogenesis, since younger brains are much more plastic than adult brains [55]. Specifically, Homberg et al. [56] found that adolescent rats treated with fluoxetine displayed increased immobility in the FST, with adult fluoxetine rats showing no effect of drug. Furthermore, Mason et al. [57] found that subchronic fluoxetine (10–20 mg/kg) in young rodents had no effect on alleviating depressive-like states in mice. These findings, similar to our own, illustrate an atypical effect of a drug that is supposed to elicit decreased immobility and increased swimming behaviors. We administered fluoxetine in late adolescence and early adulthood, which led to fluoxetine-induced increases in immobility and decreases in swimming behaviors, suggesting that the therapeutic effects of this drug may be age-dependent and also induced by differences in neural plasticity between young and adult rats [58;59]. Given that differential rearing alters neuroplasticity and neurogenesis [48], it is likely that fluoxetine-induced neurogenesis is altered by the environmental condition. While the effects of enrichment and fluoxetine have been compared [60;42], the effects of enrichment or isolation in combination with fluoxetine on neurogenesis have not yet been directly examined. The current results suggest that both age and environmental condition may be altering fluoxetine-induced neurogenesis.

Furthermore, our results illustrate the importance of not only dose, but regimen in eliciting the desired therapeutic effects of antidepressant compounds. Based on our findings, in order to elicit effective, antidepressant outcomes, fluoxetine should not be administered in moderately high doses (20 mg/kg, i.p.) in a subchronic manner. Doing so may result in weight loss and anorectic effects that can lead to reduced energy, producing confounding variables that may interfere with proper FST assessments. Indeed, the weight reducing [56; 61–63] and anorectic properties of fluoxetine [64] have been documented before. Fluoxetine (5 or 10 mg/kg for 14 days) can lead to significant reductions in body weight gain [65;66]. Fluoxetine (2–10 mg/kg) also has the ability to reduce sucrose intake in male rats if the drug is administered 30 min or 4 hrs before the presentation of sucrose, with this effect decreasing in potency with longer treatment-test intervals [67]. Furthermore, fluoxetine has been shown to cause maternal weight loss during pregnancy, and reduced litter sizes in female rats [68]. Very few studies to-date have examined the effects of subchronic, 20 mg/kg fluoxetine on weight loss.

Fluoxetine in the current study resulted in significant weight loss in all three environmental conditions which lasted about one week before fluoxetine rats regained their weight comparable to vehicle counterparts. In addition to this weight reduction, differential rearing appeared to moderate weight gain, as indicated by weight differences between IC and EC rats in both cohorts of the Experiment III experiment. This lends support to the literature illustrating the impact of various stressors on the attenuation of body weight gain [69]. Perhaps the stress of the FST and the stress involved in isolation rearing interact to alter, and in some cases, attenuate rates of body weight gain following experimental measures.

In conclusion, the current study adds to the existing literature regarding how certain environmental factors, specifically differential rearing, can impact the efficacy of commonly prescribed antidepressants. Based on this study, environmental enrichment has the ability to alter FST performance, fluoxetine efficacy, and post-test well-being. 20 mg/kg fluoxetine, administered subchronically, may lead to atypical effects opposite to those commonly observed in the FST. Environmental condition, dose, and regimen appear to play significant moderating roles in behavior before, during, and after the FST. As such, environmental factors should be taken into consideration not only when conducting preclinical measures of depression, but also when prescribing antidepressant medication.
